# Total Hip Arthroplasty with Bulk Femoral Head Autograft for Acetabular Reconstruction in Developmental Dysplasia of the Hip

**DOI:** 10.1155/2013/794218

**Published:** 2013-09-18

**Authors:** Fernando Claros Pizarro, Simon W. Young, Jorge H. Blacutt, Rolando Mojica, Juan C. Cruz

**Affiliations:** ^1^Department of Orthopaedic Surgery, Hospital Obrero No. 1, La Paz, Bolivia; ^2^Department of Orthopaedic Surgery, North Shore Hospital, Auckland 0740, New Zealand

## Abstract

Developmental hip dysplasia (DDH) presents considerable technical challenges to the primary arthroplasty surgeon. Autogenous bulk grafting using the femoral head has been utilised to achieve anatomic cup placement and superolateral bone coverage in these patients, but reported outcomes on this technique have been mixed with the lack of graft integration and subsequent collapse, an early cause of failures. We describe a novel technique combining the use of bulk autograft with an iliac osteotomy, which provides primary stability and direct cancellous-cancellous bone contact, optimising the environment for early osseointegration. Twenty-one hips in 21 patients with DDH underwent this technique and were followed for a mean of 8.1 years. The preoperative radiographic classification was Crowe type I in 12 hips (57%), type II in 4 hips, and type III in 5 hips, and the mean Sharp angle was 49.6° (range 42°–60°). All grafts united by year. At time of followup, there was no radiographic evidence of graft collapse or loosening. There were no reoperations. Our study has shown that this technique variation combining an iliac osteotomy with bulk autograft in cases of developmental hip dysplasia provides early stability and reliable graft incorporation, together with satisfactory clinical and radiological outcomes in the medium term. Longer term study is necessary to confirm the clinical success of this procedure.

## 1. Introduction

Developmental hip dysplasia (DDH) presents considerable technical challenges to the primary arthroplasty surgeon. Achieving correct positioning of the acetabular component and adequate bone coverage can be difficult. Harris first described the technique of bulk autogenous grafting to achieve superolateral bone coverage in 1977, and while early-to-midterm results were promising [1], longer term outcomes have been mixed. In Harris's series, 21% of patients had radiographic evidence of loosening at seven years [2], and outcomes at mean sixteen years showed a high rate of acetabular failure due to component loosening and graft collapse [3]. Recently, however, more favourable long-term outcomes have been reported in DDH patients utilizing bulk autografts with both cemented [4–6] and uncemented [7] implants.

Achieving union and stability of the autogenous graft have been identified as key determinants of a successful outcome with this technique [8]. Bulk autogenous grafts are known to incorporate slowly and often incompletely, [9] limiting their ability to respond to stresses under cyclic loading. The main factors affecting incorporation of the graft are stability of the construct and host-graft bone contact [1, 8]. We describe a novel technique combining the use of bulk autograft with an iliac osteotomy which provides primary stability and optimises direct cancellous-cancellous bone contact and report mid-term results on 21 patients.

## 2. Patients and Methods

During the period 1998–2001, 21 hips in 21 patients were managed with autologous bone graft in combination with an iliac osteotomy at our institution. Patients with a minimum 7-year followup were eligible for inclusion in the study. There were 3 men and 18 women in the study group, and the average age at the time of surgery was 50.1 years (range 26–77). The diagnosis in all patients was DDH, with a defective acetabular roof which would lead to uncovering of the acetabular component when placed in the desired position without augmentation.

Preoperative radiographic planning with use of transparent overlays and socket templates is first performed to evaluate the position of the socket and autograft and potential coverage. A posterior approach was used, with the short external rotators divided at their insertion and reflected to protect the sciatic nerve. The femoral head was resected and the acetabulum was exposed. A full capsulectomy was performed, and a pin inserted above the acetabulum and the distance to a fixed point on the greater trochanter measured to give an estimate of leg lengthening once trial components were placed. The acetabulum was sequentially reamed with the aim of positioning the socket at the level of the true acetabulum.

**Figure 1:**
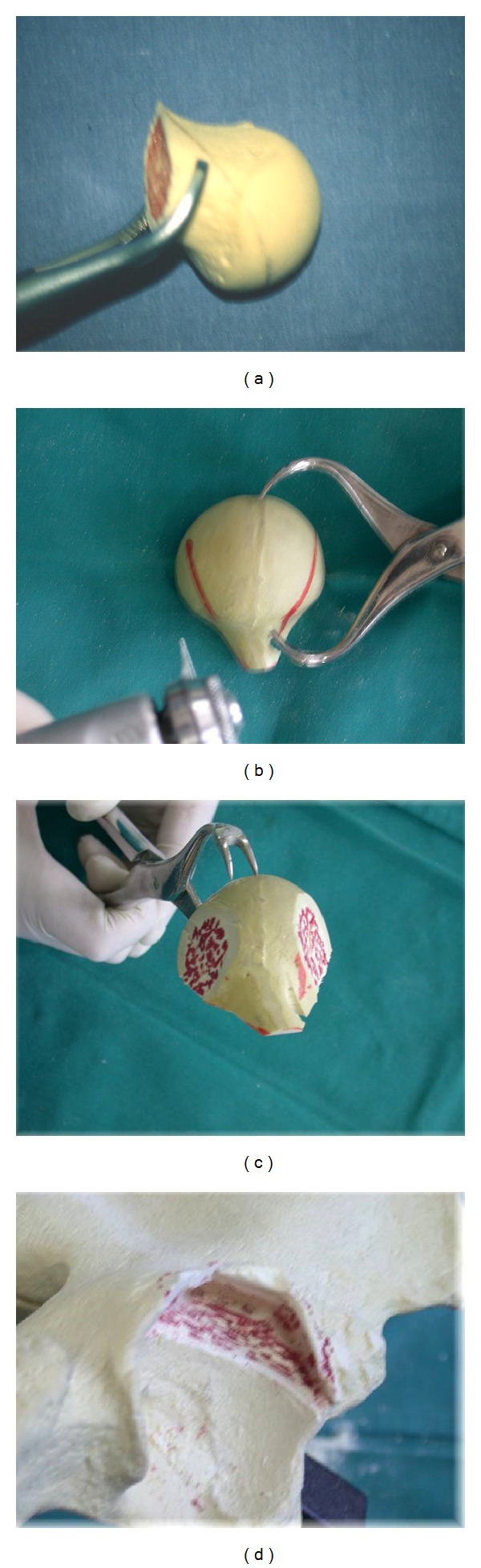
Model demonstrating preparation of the graft and ileum. Note the bevelled edges to enable impaction and primary press fit of the graft.

**Figure 2:**
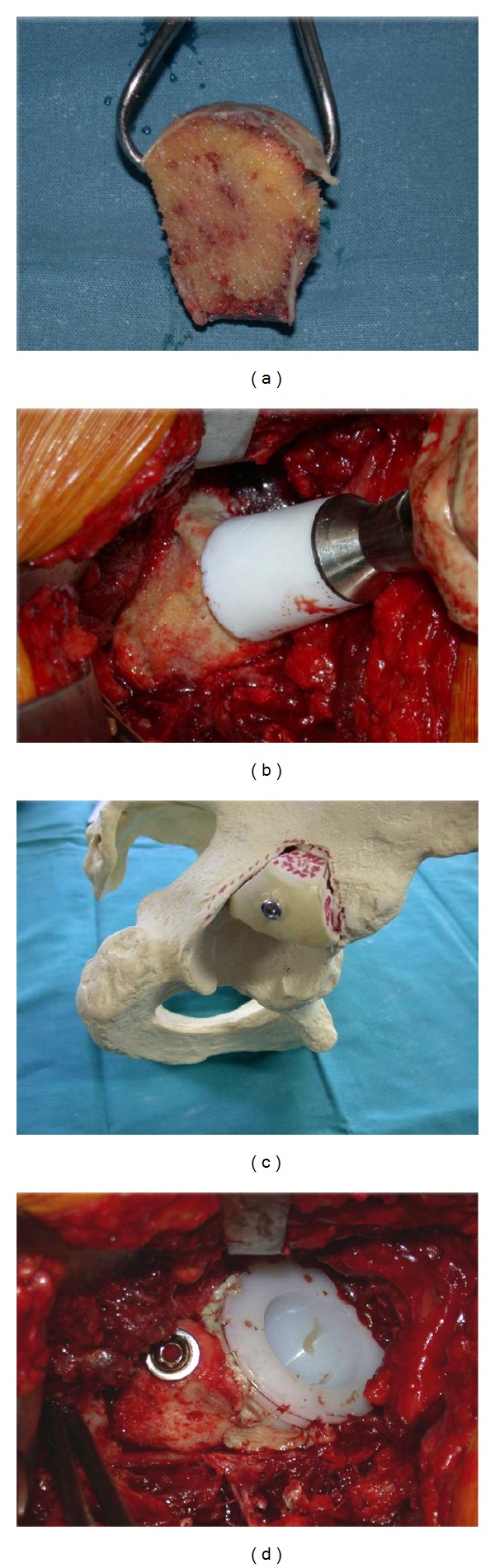
Graft before impaction and fixation in situ. Note the large cancellous bed of the graft which contacts the ileum and fixation of the graft through the sclerotic subchondral bone.

At the base of the deficient superolateral portion of the true acetabulum, a three-sided chamfered osteotomy is performed in the ileum and a wedge of bone is removed. The resected femoral head is then used as a graft with the base of the neck cut into a wedge matching the chamfer on the iliac osteotomy (Figures 1 and 2). A tight fit is achieved by fine adjustments to the graft with a saw, and this is used as a stem to place the graft into the site of the iliac osteotomy. The graft is impacted into place before being finally secured with partially threaded screws with or without washers. This construct then is reamed in the usual way, and reamings are packed into any minor defects that remain. After a saline wash, all the polyethylene cup (Lubinus, Waldemar Link, Hamburg, Germany) was then cemented in the desired position, aiming to achieve an inclination angle of 45 and 10–30 degrees of anteversion. The femoral canal was then broached and sequentially reamed before a collared monoblock implant (Lubinus SP-II, Waldemar Link, Hamburg, Germany) was cemented in situ. A 28 mm cobalt chrome head was used. Patients were allowed to weight bear immediately postoperatively.

### 2.1. Radiographic Analysis

Radiographs taken preoperatively and postoperatively at 1, 3, 5, 7, and 9 years were assessed by two observers (SWY and FC). Preoperative radiographs were classified according to the system of Crowe et al. [10] and the acetabular angle of Sharp [11] was measured. Postoperatively, the inclination angle of the socket was measured in relation to Kohler's line, and the amount of coverage of the socket was expressed as a percentage of the horizontal distance between the most medial point and the most lateral edge of the socket [3, 6]. Graft union was assessed by observing disappearance of the graft-host bone interface and the appearance of bridging trabeculae. Heterotopic ossification was classified according to the system of Brooker et al. [12]. The horizontal (distance from the inferior point of the teardrop) and vertical (distance from the interteardrop line) locations of the cup were measured as described by Russotti et al. [13]. Resorption of the graft was assessed at each follow-up interval, and the cup-bone interface was assessed based on the zones of DeLee and Charnley [14]. Loosening the cup was assessed based on the criteria of Mulroy and Harris [15], with vertical or horizontal migration >2 mm or cement fracture, inclination change >4 degrees, or a continuous radiolucent line >1 mm or noncontinuous radiolucency >2 mm at the acetabular cement-bone interface considered evidence of loosening. 

## 3. Results

The preoperative radiographic classification was Crowe type I in 12 hips (57%), type II in 4 hips, and type III in 5 hips, and the mean Sharp angle was 49.6° (range 42–60°). The mean duration of followup was 8.1 years (range 7–10.1 years). No patients were lost to followup.

Immediate postoperative radiographs showed a mean acetabular inclination angle of 43° (range 28–62°), and the average coverage of the component by graft was 40.2% (range 24–60%). The mean horizontal location of the hip centre was 35 mm (range 22–42 mm) lateral to the teardrop. The mean height of the hip centre was 31 mm (range 20–56 mm) vertically from the interteardrop line. Russotti and Harris [13] defined proximal placement of the socket as ≥35 mm of vertical displacement, and according to these criteria six patients had proximal placement of the socket. 

**Figure 3:**
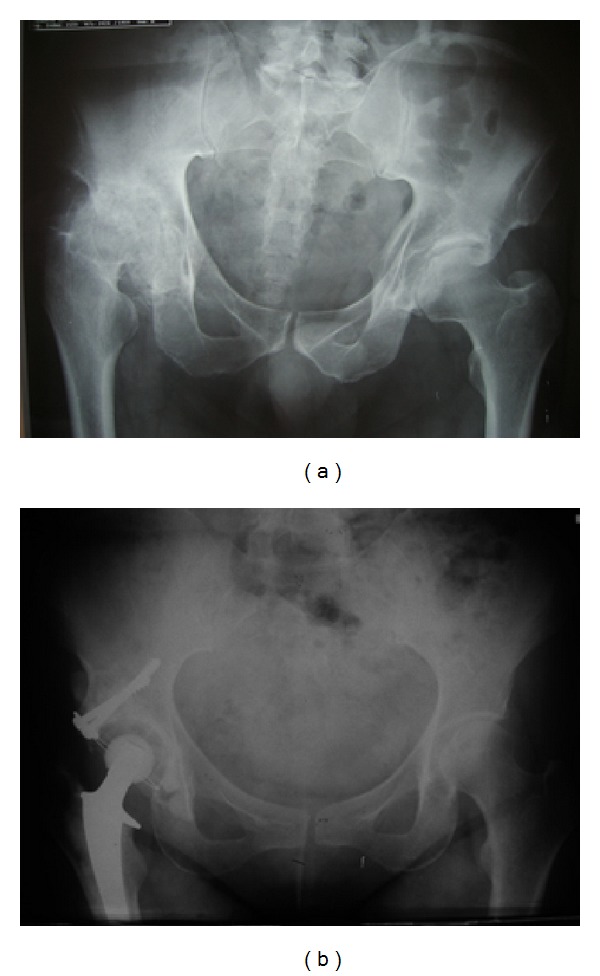
Preoperative (a) and seven-year postoperative (b) X-rays illustrating positioning of the graft.

**Figure 4:**
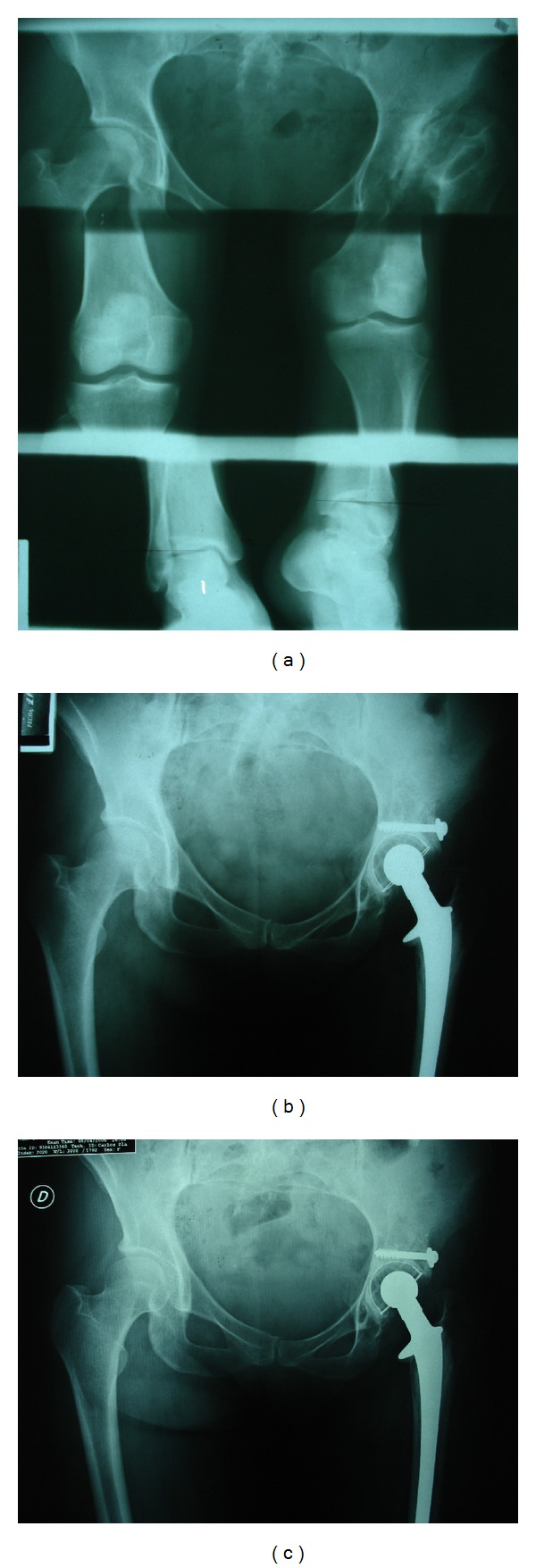
Preoperative (a) and postoperative X-rays at 1-year (b) and at 8-year (c) followup illustrating restoration of hip centre of rotation and osteointegration of the graft.

All grafts were united by one year on followup radiographs. At latest followup, all hips were functioning well with no clinical signs of loosening and no revisions had been performed (Figures 3 and 4). There was no discernible graft resorption or collapse and no patient had acetabular loosening according to our criteria. No patient had vertical or horizontal migration >2 mm or a change in the inclination angle >3°. Two patients had noncontinuous radiolucent lines of <1 mm visible at seven-year followup which were not apparent at 3 or five year followup. 

Postoperative complications included transient neuropraxias in 3 patients, an episode of dislocation in one patient which was treated with closed reduction. Two patients showed evidence of heterotopic ossification, one Brooker stage I and one Brooker stage II. There were no infective complications. 

## 4. Discussion

A combination of a high local incidence of DDH in Bolivia, possibly related to both genetic and environmental factors including swaddling of infants, together with limited primary care facilities in childhood leads to a high proportion of patients presenting in the 3rd or 4th decade of life with degenerative hip disease.

Developmental dysplasia of the hip presents considerable difficulties in restoration of the acetabular anatomy and achieving coverage of the acetabular component, particularly in the superolateral region. Strategies to address this problem include proximal positioning of a smaller cup [13, 16] penetration of the medial wall (protrusio technique) [17, 18], an iliac sliding graft [19], and lateral bulk grafting [1, 4–6] with either autogenous or allogenic bone. While all of these techniques have reported satisfactory medium term results, bulk autogenous grafting has been favoured by many authors as it allows more anatomic cup placement and provides early structural support to the acetabular component, and there is a ready availability of autogenous graft in the form of the resected femoral head. A further advantage is the augmentation of pelvic bone stock in case of subsequent revision [20], an important consideration given the often early age of onset of secondary degenerative change in this population group. 

Published results of bulk autograft in DDH have been variable, probably because of differences in patient selection, the severity of dysplasia, bone quality, and the technique of bone grafting and components used [20]. Harris et al. reported good early results in 27 hips in 22 patients [1] but subsequently reported a combined clinical and radiographic rate of 60% at 16 years with failures due to graft resorption and collapse [3]. In contrast, Kobayashi et al. [6] reported on 37 hips at a mean of 19 years after cemented arthroplasty with 100% survival of the socket, and other authors have also reported favourable outcomes [4, 5, 7, 20–22].

Previous studies have identified the two most important factors in graft incorporation as the host-graft bone contact and the stability of the graft [1, 8, 9, 19]. Harris originally described “scoring” of the lateral cortex of the ileum before directly bolting the curvature of the femoral head against the ileum. Later authors have described using a cut surface of the femoral head [7, 22] as a graft to ensure cancellous bone is contact with the ileum. In addition, Kobayashi described preparation of the ileum with multiple drill holes [6] to obtain a bleeding bone bed to encourage graft incorporation. 

In this study, we describe a technique of graft and iliac preparation which maximises the area of cancellous-cancellous bone contact between graft and host bone. In addition, the bevelled nature of the osteotomy allows impaction of the graft and gives primary stability, meaning that the screws provide supplementary fixation only. This optimises the biological situation for revascularisation and incorporation of the graft, which hopefully will lead to an increase in bone stock and possibly reduced resorption and failure rates in the longer term. 

Ikeuchi et al. [19] described the use of a sliding iliac graft for acetabular reconstruction in DDH patients which utilised a similar osteotomy to that in this study. They reported excellent short term outcomes in 19 patients and noted rapid incorporation of the graft secondary to intimate host-graft contact and stability and a subsequent low rate of resorption. However, the maximum thickness of the iliac graft was 14 mm, limiting the technique in more severe cases of dysplasia. Our method retains the advantages of host-graft contact and stability without limitation in available graft size. 

There are a number of limitations to our study. Firstly, we used cemented implants only, due to availability and cost in the country where the study was performed. While early series on bulk autograft also used cemented implants [1], in recent years there has been a move to uncemented components particularly on the acetabular side. Secondly we lack pre- and postoperative clinical outcome scores, partly due to the lack of cultural and language appropriate validated scoring instruments. 

In conclusion, our study has shown that this technique variation combining an iliac osteotomy with bulk autograft in cases of developmental hip dysplasia provides early stability and reliable graft incorporation, together with satisfactory clinical and radiological outcomes in the medium term. Longer term followup is necessary to confirm the clinical success of this procedure.
